# The complete chloroplast genome sequence of *Zelkova sinica* and phylogenetic implications in Rosales

**DOI:** 10.1080/23802359.2021.1987168

**Published:** 2021-10-07

**Authors:** Xinyi Liu, Rui Li, Shuyun Wang, Luxian Liu

**Affiliations:** a Key laboratory of Plant Stress Biology, School of Life Sciences, Henan University, Kaifeng, China; b School of Pharmacy, Henan University, Kaifeng, China

**Keywords:** *Zelkova sinica*, genome skimming, chloroplast genome, phylogeny inference

## Abstract

*Zelkova sinica* is a popular landscape plant in China because of its wide adaptation, strong disease resistance, large crown and beautiful fall color. Here, we assembled the complete chloroplast (cp) genome of *Z. sinica* based on genome skimming data. The cp genome is 158,924 bp in length including two copies of inverted region (IR, 26,427 bp) separated by the large single copy (LSC, 87,318 bp) and small single copy (SSC, 18,752 bp) regions. It encodes 111 unique genes, containing 77 protein-coding genes, 30 tRNA genes, and 4 rRNA genes, with 18 duplicated genes in the IR regions. Phylogenetic analysis shows that *Z. sinica* is sister to *Z. schneideriana* in Ulmaceae family.

*Zelkova sinica* Schneid., which belongs to Ulmaceae, is a species naturally distributed in the northwestern region in China (Chen and Huang 1999). *Z. sinica* is one of the most popular landscape plants in China due to its adaptability to varied environmental conditions, strong disease resistance, outstanding crown, and beautiful fall color (Jin and He 2005). Because of its beautiful and durable texture, the wood of this species has been widely used for making furniture (Jin and Hu 2012). Nowadays, *Z. sinica* is considered as a rare and endangered species because of a sharp decline of its distribution range, which is a consequence of overexploitation of the wild plants for commercial purposes and inefficient propagation methods (Fu and Jin 1992). However, researches regarding its genetic background are extremely scare. In this study, we used genome skimming data for assembling the complete cp genome of *Z. sinica*. The genome sequence was registered into GenBank with the accession number MW850753.

One individual of *Z. sinica* was sampled at Jinming Campus of Henan University (114°18′29.84″E, 34°49′13.53″N), Kaifeng, Henan, China. Fresh leaves were collected for genomic DNA extraction using modified CTAB (Cetyltrimethylammonium Bromide) reagents (Plant DNAzol, Shanghai, China) according to the manufacturer’s protocol. A voucher specimen (Luxian Liu *LLX2021032506*) was deposited at the Herbarium of Henan University. High quality DNA was sheared to yield fragments less than or equal to 800 bp, and the 500 bp short-insert length paired-end library was prepared and sequenced on an Illumina HiSeq X10 to obtain reads of 150 bp at Beijing Genomics Institute (BGI, Wuhan, China). The raw data was screened by quality with Phred score <30 and assembled into contigs using the CLC Genomic Workbench (CLC Inc. Aarhus, Denmark). The complete cp genome of *Z. sinica* was constructed and annotated using the software Geneious R11 (Biomatters, Auckland, New Zealand) following description in Liu et al. (2017**)** and Liu et al. (2020**)** with *Z. serrata* (GenBank accession number: MG717940) as a reference. Phylogenetic tree for 23 whole cp genome sequences of Rosales was constructed using maximum likelihood (ML) method implemented in RAxML-HPC v8.2.12 on the CIPRES cluster (Miller et al. 2010) with *Cucumis sativus* and *Cucumis melo* as outgroups. 1000 bootstrap iterations were conducted with other parameters using the default settings and GTR + G was determined by the software jModel Test v2.1.4 (Posada 2008) as the best-fit nucleotide substitution model.

There were 118,113,846 paired-end reads received for *Z. sinica*, and 56,519,152 reads were removed from the raw data after trimming low quality sequences. The cp genome of *Z. sinica* was 158,924 bp in length, and had a typical quadripartite structure consisting of an 87,318 bp large single copy region (LSC), a 18,752 bp small copy region (SSC) and two 26,427 bp inverted repeats (IRs). Within the genome of *Z. sinica*, there are 111 unique genes, including 77 protein-coding genes, 30 tRNA genes, and 4 rRNA genes, additionally with 18 duplicated genes in the IR regions. Among the 111 genes, 6 tRNA genes, and 9 protein-coding genes contain a single intron, and three genes (*rps*12, *clp*P, and *ycf*3) contain two introns. The overall GC content of the total length, LSC, SSC, and IR regions is 35.6, 33.0, 28.3 and 42.3%, respectively. The phylogeny revealed that the three representative genera of Ulmaceae, including *Zelkova, Ulmus* and *Hemiptelea,* formed a full supported clade, and *Z. sinica* is sister to *Z. schneideriana* within *Zelkova* (Figure 1). The result revealed in this study is consistent with that of Chase et al. (2016**)** except for the position of Barbeyaceae, which is sister to the families including Dirachmaceae, Rhamnaceae, and Elaeagnaceae in the latter one.

**Figure 1.**
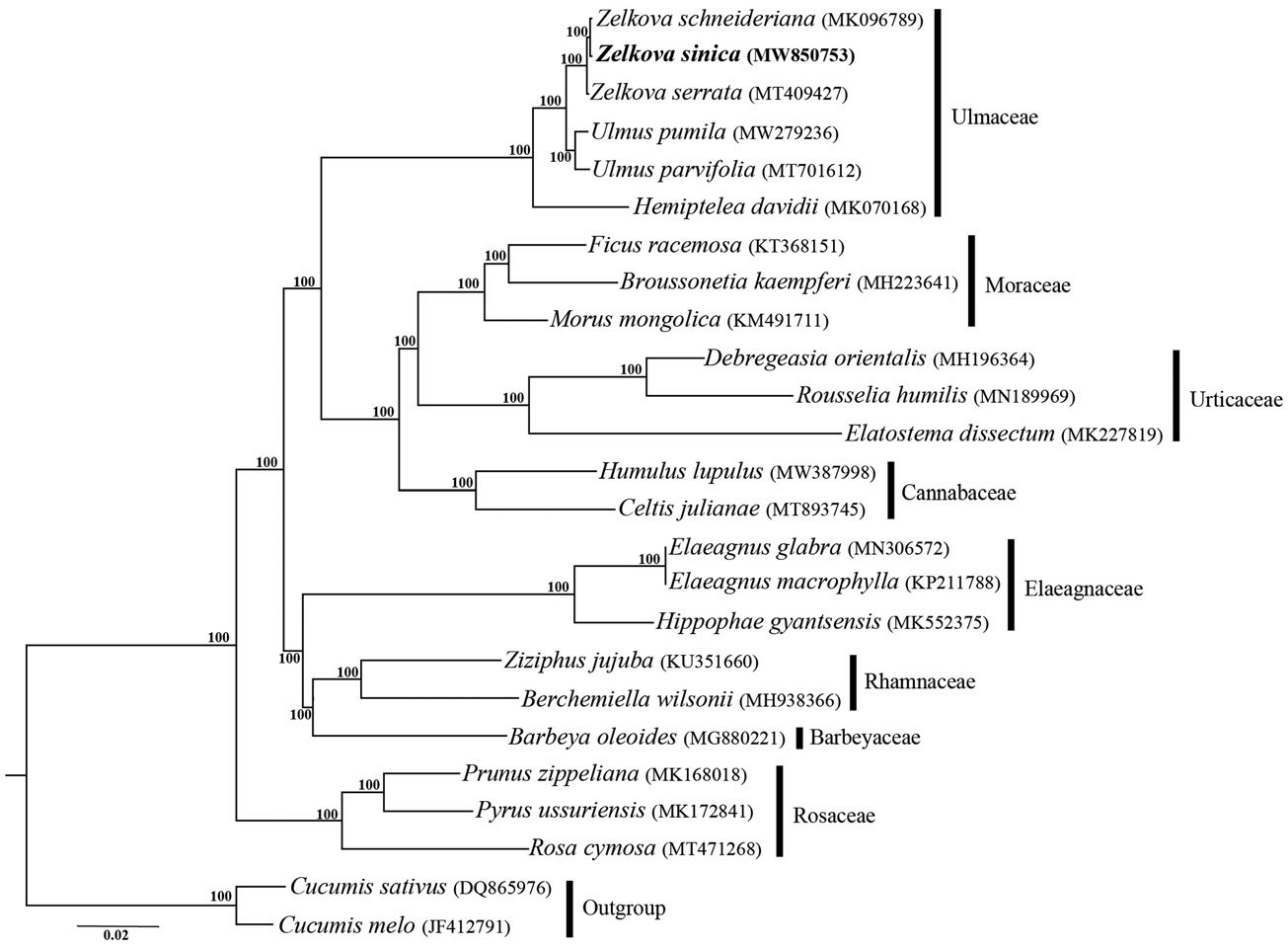
Phylogenetic tree reconstruction of Rosales using maximum likelihood (ML) based on whole chloroplast genome sequences. Numbers above the lines represent ML bootstrap values.

## Data Availability

The genome sequence data that support the findings of this study are openly available in GenBank of NCBI at https://www.ncbi.nlm.nih.gov/ under the accession no. MW850753. The associated Bio-Project, SRA and Bio-Sample numbers of the raw sequence data for assembling the cp genome are PRJNA735441, SRR14741978, and SAMN19582534, respectively.
